# Color contrast adaptation and compensation in color deficiencies

**DOI:** 10.1167/jov.25.10.17

**Published:** 2025-08-29

**Authors:** Fatemeh Basim, Erin Goddard, Yueran Yang, Michael A. Webster

**Affiliations:** 1 Graduate Program in Integrative Neuroscience, University of Nevada Reno, Reno, NV, USA; 2 School of Psychology, University of New South Wales, Sydney, Australia; 3 Department of Psychology, University of Nevada Reno, Reno, NV, USA

**Keywords:** color vision, color deficiencies, compensation, adaptation, plasticity

## Abstract

Anomalous trichromacy (AT) results from a reduced spectral separation between the L and M cone photopigments. This leads to smaller differential responses in the L and M cones and thus lower sensitivity to the colors signaled by the LvsM difference. Despite this, for stimuli above threshold, many color-anomalous observers report color experiences that resemble those of color-normal individuals, suggesting some form of perceptual compensation for their sensitivity losses. The nature and sites of this compensation remain uncertain, and may reflect many levels, from early sensory mechanisms to later post-perceptual processes. To assess potential sensory-level compensation, we compared the aftereffects of adaptation to chromatic contrast in 15 color-normal and 15 color-anomalous observers (10 deutan, 5 protan). Without compensation, the same adapting contrast should produce weaker adaptation effects in anomalous observers, because the same physical adaptor is a lower multiple of their threshold sensitivity. We quantified this prediction in color-normals by rescaling the LvsM contrasts to simulate the sensitivity losses. Although protan observers showed mixed results, the deutan observers exhibited adaptation effects that exceeded the predictions based on their threshold sensitivities, indicating partial compensation for the reduced LvsM signals. These findings are consistent with a post-receptoral sensory gain in contrast processing that compensates for the weaker LvsM cone signals available to anomalous observers, and could reflect a general normalization of contrast coding to match the color gamut of the observer's environment.

## Introduction

Trichromatic color vision depends on comparing the responses in three classes of cone photoreceptor that have peak sensitivity to long (L), medium (M), or short (S) wavelengths. In individuals with normal color vision (CN), the peak separation between the L and M cones is roughly 25 nm. Anomalous trichromacy (AT) is a common inherited color vision deficiency affecting about 6% of males of European descent, and results from alterations in the genes coding the L and M photopigments (Neitz & Neitz, 2011). The altered pigments reduce the spectral differences between the normal and anomalous L and M cones, with peak differences ranging from 12 nm to as little as 1 nm. In deuteranomalous observers, the peak of the anomalous pigment is shifted toward the normal L pigment, whereas in protanomalous observers the anomalous pigment is shifted toward the normal M cone. In both cases the reduced spectral separation results in smaller signals conveyed by the difference in the L and M responses (LvsM) and is typically manifest as weaker discrimination for LvsM chromatic differences.

Despite this limitation in the initial sensory representation, AT observers often report color experiences that appear subjectively similar to those of CN individuals (Bosten, 2019; Isherwood, Joyce, Parthasarathy, & Webster, 2020). For example, ATs and even dichromats can often name colors similarly to CNs (Bonnardel, 2006; Lindsey, Brown, & Hutchinson, 2021), and their judgments of color salience or similarity can be stronger or even comparable to CNs despite their poorer discrimination at threshold (Boehm, MacLeod, & Bosten, 2014; Regan & Mollon, 1997). Such results have led a number of researchers to suggest that post-receptoral processing of the cone signals may adjust to compensate for the weaker differential inputs from the cones. However, the sites and nature of such adjustments remain uncertain, and could potentially arise at many stages, from early in the visual system (where for example the gain of retinal neurons encoding the LvsM signals might be amplified), to how the signals are represented in early visual cortex, to late stages (e.g., reflecting how the signals are decoded to interpret or categorize color) (Isherwood et al., 2020). One of the “magics” of psychophysics is the ability to use behavioral assessments to probe these different stages and their properties, and compensation for color deficiencies has been explored with a variety of psychophysical techniques, from discrimination to reaction times to perceptual scaling and naming (Basim et al., 2025; Boehm, Bosten, & MacLeod, 2021; Boehm et al., 2014; Emery, Kuppuswamy Parthasarathy, Joyce, & Webster, 2021; Knoblauch, Marsh-Armstrong, & Werner, 2020; Lindsey et al., 2021; Regan & Mollon, 1997; Robinson, Bosten, & MacLeod, 2023; Vanston, Tregillus, Webster, & Crognale, 2021).

In this study, we examined color compensation by using visual adaptation, or the changes in sensitivity and appearance that result from prior exposure to stimuli. Adaptation occurs throughout the visual stream, affecting most if not all perceptual attributes (Clifford et al., 2007; Webster, 2015). It has been called “the psychologist's electrode” (Frisby, 1979), because by choosing the appropriate stimuli and measurements, the adaptation can be used to target and dissect the visual representations at different processing stages (Graham, 1989; Webster, 2011). Harnessing adaptation to probe perceptual processing was one of the hallmarks of Randolph Blake's research, which this special issue commemorates (e.g., Alais & Blake, 1999; Alais, Cass, O'Shea, & Blake, 2010; Blake & Fox, 1974; Blake, Overton, & Lema-Stern, 1981; Blake, Tadin, Sobel, Raissian, & Chong, 2006; Yang, Hong, & Blake, 2010). In color vision, several distinct types of adaptation have been documented and tied to different levels and mechanisms (Webster, 1996). Adaptation begins with independent sensitivity changes in the receptors, which adjust to changes in the ambient light level (Smithson, 2005). Characterizations of “second-site” adaptation were fundamental in revealing post-receptoral mechanisms that receive opposing signals from the cones (Pugh & Mollon, 1979). Studies of “color contrast” adaptation paralleled measurements of pattern-selective adaptation in identifying the representation of luminance or chromatic contrast. Specifically, just as spatial contrast (e.g. in gratings) was used to characterize selectivity for stimulus properties such as spatial frequency or orientation (Graham, 1989), “color contrast” (e.g., flickering or varying color along different directions in color space) has helped reveal the channel structure underlying cortical color coding (Krauskopf, Williams, & Heeley, 1982; Webster & Mollon, 1991). Dissociable mechanisms of both color and contrast adaptation have also been distinguished based on the time course of the adjustments, with fast mechanisms reflecting rapid neural gain adjustments and slower mechanisms reflecting longer-term recalibration of perceptual norms. (Bao & Engel, 2012; Belmore & Shevell, 2008; Delahunt, Webster, Ma, & Werner, 2004; Neitz, Carroll, Yamauchi, Neitz, & Williams, 2002; Webster & Leonard, 2008).

Here we focus on chromatic contrast adaptation in color-normal and anomalous observers. In a highly influential study, Krauskopf, Williams, and Heeley (Krauskopf et al., 1982) measured sensitivity to a color change from a white background after adapting to temporal modulations in color along different directions in color space. The adaptation (which they referred to as habituation) selectively elevates thresholds for color changes along the adapting axis, and in particular, produces strongly selective threshold changes for stimuli defined by the LvsM or SvsLM axes. These results prompted the identification of these dimensions as the “cardinal” axes of color coding, and correspond to the primary opponent dimensions along which color signals are conveyed in the retina and geniculate (Derrington, Krauskopf, & Lennie, 1984). However, several lines of evidence suggest that the adaptation to chromatic contrast primarily has a cortical locus. For example, the adaptation shows interocular transfer (Webster & Mollon, 1994) and is also selective for the spatial frequency or orientation of the stimuli (Bradley, Switkes, & De Valois, 1988; Clifford, Spehar, Solomon, Martin, & Zaidi, 2003). The adaptation can also be selective for multiple directions in color space, especially when the effects are measured on suprathreshold appearance (Krauskopf, Williams, Mandler, & Brown, 1986; Webster & Mollon, 1991; Webster & Mollon, 1994). This selectivity is consistent with the elaboration of “higher-order” color mechanisms in color coding (Krauskopf et al., 1986), and with the broader diversity of color preferences found in primary visual cortex compared to the LGN (Lennie, Krauskopf, & Sclar, 1990; Li, Garg, Zhang, Rashid, & Callaway, 2022).

In our study we exploited the adaptation paradigm developed by Krauskopf et al. to compare the strength of the adaptation in color-normal and anomalous observers. In particular, we leveraged the finding that the strength of color contrast adaptation increases with the level of the adapting contrast (Krauskopf et al., 1982; Webster & Mollon, 1997). Because ATs have weaker sensitivity to LvsM contrasts, without compensation they should have weaker adaptation to the LvsM signals, because the same physical LvsM stimulus is a lower multiple of their threshold sensitivity. The predicted magnitude of this effect depends on how the threshold changes vary with adapting contrast. To directly assess this, we measured the actual change in adaptation in color-normal observers when the LvsM contrasts were scaled to mimic different levels of sensitivity loss, and then compared these predicted settings to the magnitude of adaptation in the color-anomalous observers. The results are consistent with a number of other studies in suggesting that many anomalous observers show partial but incomplete compensation for their altered cone sensitivities, and psychophysically tie the compensation to sensory gain changes occurring at or before the early visual cortex.

## Methods

### Participants

The experiment was carried out at two separate locations, University of Nevada, Reno (UNR), and the University of New South Wales (UNSW) in Sydney. We used identical display equipment and testing protocols in both locations. Conducting the study across both sites allowed for more efficient participant recruitment. We tested a total of 30 individuals (14 females, 16 males), with 11 completing the study at UNR and 19 at UNSW. Ages ranged from 19 to 56 years (*M* = 28.04, *SD* = 9.80). Participants were grouped based on color vision classification: normal trichromats (five at UNR, seven at UNSW), deuteranomalous observers (three at UNR, seven at UNSW), and protanomalous observers (three at UNR, two at UNSW). Color vision status was evaluated by using Rayleigh matching on an Oculus anomaloscope. All participants provided informed consent, and followed protocols approved by the IRBs at each university. The current study was part of a battery of assessments comparing color vision across a range of perceptual tasks. One participant classified as CN was excluded from the current study based on uncharacteristically high contrast thresholds for all of the axes tested (LvsM, SvsLM, and luminance) (see Basim et al., 2025). Following previous studies (e.g., Boehm et al., 2021; Boehm et al., 2014; Knoblauch et al., 2020; Regan & Mollon, 1997) we analyze the results for deuteranomalous and protanomalous observers separately, but note that there are no a priori theoretical grounds for expecting differences in compensation between the two groups.

### Stimuli and apparatus

Stimuli were shown on Display++ monitors (Cambridge Research Systems), calibrated using a Photo Research PR655 spectroradiometer. The stimulus consisted of a 2 × 2 array of uniform square fields, each measuring 4.76° × 4.76° and separated by 0.34°. The full array spanned 9.85° × 9.85° and appeared centered on a 33.7° × 49.4° background. Each square had a narrow black border to separate it from the background. These outlines remained visible throughout the trial.

The colors in the fields were chosen to vary the LvsM signal relative to the gray adapting background, based on the Stockman and Sharpe cone fundamentals (Stockman & Sharpe, 2000). The LvsM contrast was specified by a variant of the space developed by MacLeod–Boynton (MacLeod & Boynton, 1979), which directly represents chromatic stimuli according to the LvsM (L/(L+M)) and SvsLM (S/(L+M)) cardinal axes at constant luminance (L+M). As in the Derrington-Krauskopf-Lennie variant of the space (Derrington et al, 1984), we centered the color space on a gray point (corresponding to D65; CIE 1931 x, y = 0.313, 0.329), which served as both the background chromaticity and the neutral reference for all stimuli, and scaled the LvsM signals a priori so that a value of 1 corresponded roughly to the normal threshold contrast for detecting the change from the background (and represented a contrast change of 0.057% in the L cones and −0.133% in the M cones).

The contrast units in our space are related to the MacLeod–Boynton coordinates by the following equations:

$$\begin{array}{ccc} LvsM & = & 2500\times (l_{mb}-0.6981)\\ SvsLM & = & 5000\times (s_{mb}-0.02065) \end{array}$$

where 0.6981 and 0.02065 represent the MacLeod-Boynton L/L + M) and S/(L + M) values of the D65 gray point. Note that in the present experiments, the SvsLM value remained constant at the background level, and thus only the LvsM value varied.

### Procedure

Participants viewed the display binocularly from a distance of 60 cm in a darkened room and responded using a handheld numeric keypad. A central fixation cross remained visible throughout the session, and participants were instructed to maintain fixation at all times. Prior to the main experiment, each participant completed a minimum-motion task to determine individual luminance nulls along the LvsM and SvsLM axes (Cavanagh, MacLeod, & Anstis, 1987). The stimuli were 0.95 c/° gratings with a chromatic contrast of 80 units along each axis, and were alternated at 2 Hz with an achromatic grating of 20% Michelson contrast. The gratings were shown in all four of the fields and the luminance contrast of the chromatic grating was varied in a staircase to estimate the motion null. Participants made four settings for each axis, and the mean setting was then used to adjust the stimuli for equiluminance for each observer.

**Figure 1.**
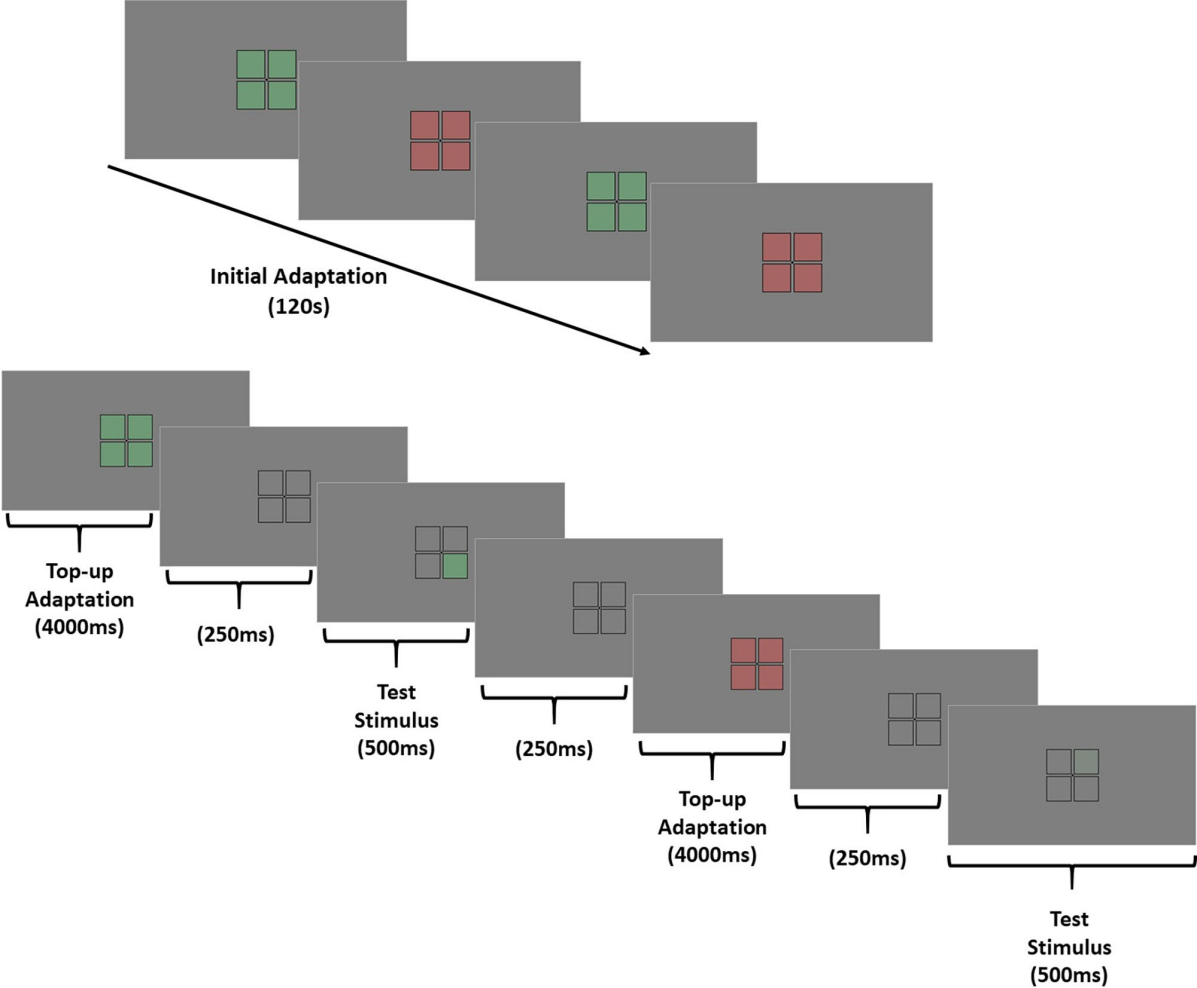
Schematic of the experimental setup and trial sequence. (Top) Initial adaptation phase lasting 120 seconds, during which all four fields were sinusoidally flickered in chromaticity at 1 Hz. (Bottom) The testing sequence included an adaptation top-up (four seconds), pretest interstimulus interval (250 ms), test stimulus presentation (500 ms), and post-test interstimulus interval (250 ms). This sequence was repeated until the staircase terminated.

Thresholds for detecting the LvsM stimulus were measured with a spatial 4AFC staircase. For the pre-adapt settings, the observer first adapted to the gray stimulus for 15 seconds, and each test presentation was followed by 2000 ms of readaptation to the neutral background. On each trial, one of four square fields briefly changed color for 500 ms, accompanied by a beep. Participants identified the location of the change using a handheld keypad. The staircase varied the contrast in increments of 0.2 log units (reduced to 0.1 after three reversals), and thresholds were computed from the average of the final nine of the 13 staircase reversals. The threshold settings were repeated three times for each of the LvsM and −LvsM directions in the pre-adapt phase, with the order of presentations counterbalanced across repetitions. In a second set of six runs, the observers first viewed sinusoidal flicker at 1 Hz for 120 seconds. The flicker modulated the LvsM signal over a range of ±80 units, and was shown in all four fields. The initial adaptation was followed by the same threshold task, but interleaved with four-second top-up adaptation between each trial (with a gray field shown for 250 ms between each test and adapt stimulus). A schematic of the testing sequence is shown in Figure 1. Measurements were collected over two sessions on different days. In each, the observer first completed three pre-adapt settings and then three adapt settings. In the first session, they also completed two brief practice runs before the measurements, one without adaptation and one with a shortened 10-second adaptation period, to familiarize themselves with the task. Results reported are based on the average of the six total settings for the pre-adapt and adapt conditions for each individual.

For color-normal observers only, we conducted a second auxiliary study to measure thresholds and adaptation after scaling the LvsM contrasts to simulate different levels of LvsM sensitivity loss. The scaling reduced the contrast of both the test and adapt stimuli by a common factor corresponding to sensitivity loss values of approximately 1.00, 2.50, 6.25, and 15.15, where a sensitivity loss of 1.00 indicates no reduction and 15.15 corresponds to the greatest reduction in contrast. During the session, the observer first made two pre-adapt settings using the same staircase procedure. They then made settings two times after adapting to each contrast level, ordered from lowest to highest contrast to reduce potential carry-over of the adaptation from a previous run. This sequence was repeated in two sessions on different days for a total of four repeated runs per condition. Results reported are again based on the average of the thresholds for each condition for each observer.

## Results

**Figure 2.**
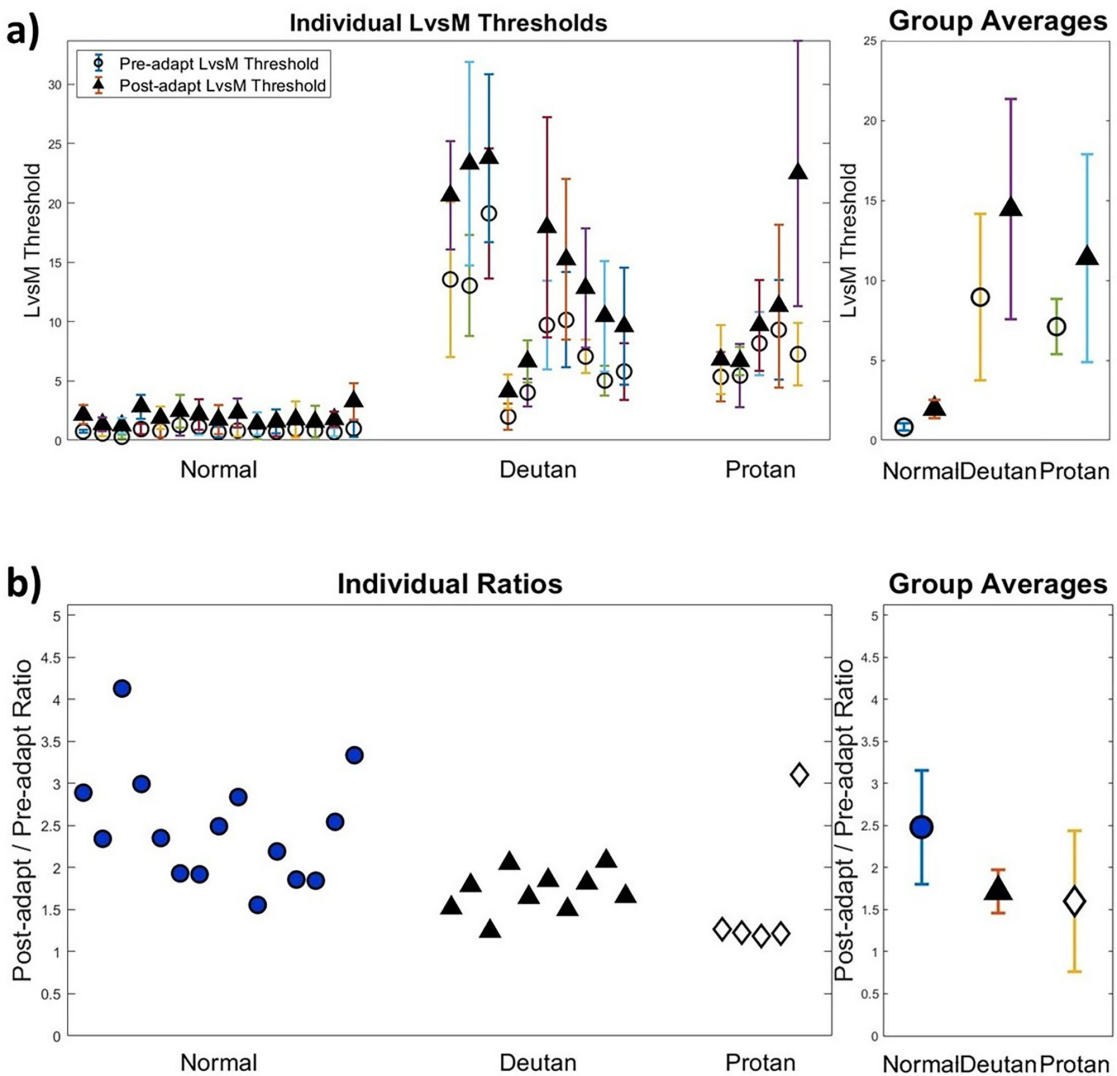
(**a**) Thresholds for detecting the LvsM color change before (circles) or after adapting (triangles) adapting to LvsM contrast. Left panel plots the threshold for individual color-normal, deutan, or protan observers. Right panel shows the mean thresholds for each group ± 1 *SD*. (**b**) Ratio of the post-adapt to pre-adapt thresholds for each observer and the corresponding group means.

Figure 2a plots the individual LvsM thresholds for the different observers before or after adaptation. For the ATs, the pre-adapt thresholds averaged 10.85 (deutan) or 8.61 (protan) times higher than the average CN value. Mean thresholds for the SvsLM and luminance axes did not significantly differ (reported in Basim et al., 2025). Prior adaptation to the LvsM contrast significantly increased thresholds in both the Deuteranomalous group (*t*(9) = 6.95, *p* = 6.66 × 10^−^^5^) and the Normal (non-scaled) group (*t*(14) = 9.40, *p* = 2.01 × 10^−7^), whereas the increase was not statistically significant in the Protanomalous group (*t*(4) = 1.55, *p* = 0.20). Figure 2b shows the ratio of the adapted to unadapted thresholds for each observer. For CNs this ratio averaged 2.48, whereas for the ATs the mean value was 1.72 (deutan) or 1.6 (protan). There is evident spread and overlap in the individual values across the groups, so that some of the ATs had adaptation effects comparable in magnitude to some of the CNs. The group means of the ratios were compared using a Welch's *t*-test for samples of unequal variance and were found to be significantly smaller than for the CNs (*t*(15.65) = −3.22, *p* = 0.0061). This indicates that the adaptation (at least as measured by the proportional change) was weaker on average for the AT observers.

**Figure 3.**
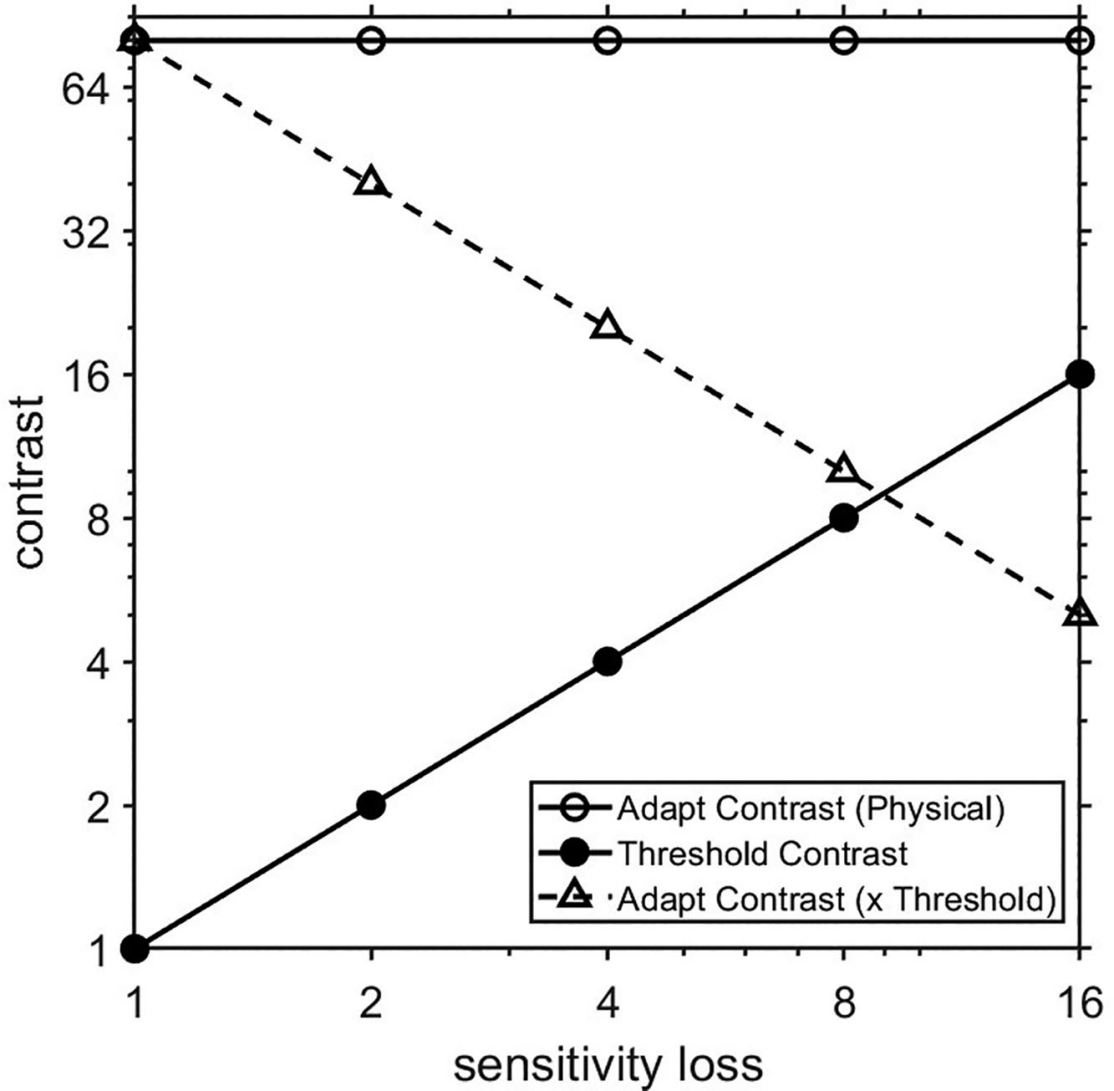
Schematic illustration of rescaling contrast to simulate sensitivity losses. As contrast is reduced, thresholds should proportionately increase (filled circles). Correspondingly, sensitivity to a fixed physical adapting contrast (unfilled circles) should decrease, because it is a lower multiple of the threshold contrast (triangles).

The smaller adaptation effects on average for the ATs suggests that compensation for the adapting contrast—if it does occur—is generally not complete. However, this does not address the converse of whether the adaptation was nevertheless stronger than predicted. As noted, to assess this, we measured how a simple rescaling of the LvsM stimulus contrast would affect thresholds and adaptation in color-normal observers, and then used this as a baseline for assessing the adaptation effects in the anomalous observers. Rescaling the LvsM contrast is roughly equivalent to the response changes that should occur in the ATs if there is a reduced separation in the peaks of their longer-wave cones, but signals are otherwise processed in the same way as in normal trichromats (Boehm et al., 2014). The consequences for the effective adapting contrast are illustrated in Figure 3. As the stimulus contrast is scaled to lower values, thresholds for detecting the contrast should increase proportionately. For example, if the contrast is two times lower, the predicted threshold should be two times higher. However, this scaling also alters sensitivity to the LvsM adapting contrast. Again for a twofold sensitivity loss, the effective adapting contrast should also decrease twofold. The net result is that as sensitivity decreases, adaptation should become weaker because it becomes a weaker stimulus for the observer. The specific change in the adaptation depends on how the strength of adaptation varies with stimulus contrast, and on nonlinearities in the response to contrast. However, rather than modeling these, we used direct measurements of the response from color-normal observers and then compared these to the observed adaptation effects in the ATs.

**Figure 4.**
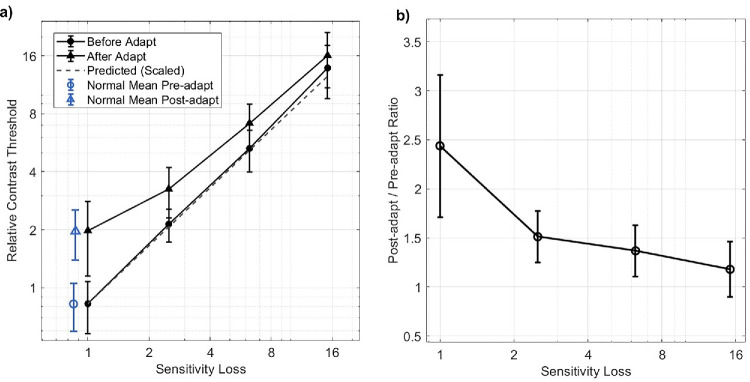
Change in adaptation magnitude with stimulus scaling for CN observers. (**a**) Measured thresholds before (circles) and after (triangles) adaptation for different scalings of the stimulus contrast. Points plot the average of the color-normal observers ±1 *SD*. Dashed line shows the predicted change in the pre-adapt thresholds from rescaling the stimulus contrast. (**b**) ratio of post- to pre-adapt contrast threshold for the different contrast scalings, ± 1 *SD*.

The effects on the thresholds before and after adaptation of rescaling the stimulus to simulate sensitivity losses are shown in Figure 4 for the 13 CNs tested in this experiment. Figure 4a shows the average thresholds before or after adaptation, for each sensitivity scaling factor. Again, the value of 1 corresponds to the original contrast (as given by the equations in Methods), whereas higher values are after reducing the default contrast by the corresponding factor. Not surprisingly, the pre-adapt thresholds increase in close proportion to the loss predicted by the rescaling (open symbols), suggesting that the average thresholds themselves were highly replicable. This is also indicated by the similarity of the unit-scaled contrast thresholds to the mean threshold for the CNs shown in Figure 2, which are replotted by the blue, unconnected symbols in Figure 4a. These values are very similar, which indicates that the different testing sequences in the two experiments did not bias the thresholds (which, for example, could have occurred because of potential differences in build-up of adaptation during the two sequences). The adapted thresholds also increase with the simulated sensitivity loss, but as expected, show the strongest adaptation at the full contrast, while dissipating at the reduced contrasts. The proportional change in adaptation strength with contrast scaling is shown in Figure 4b and provides the benchmark for comparing the strength of adaptation in the color-anomalous observers.

**Figure 5.**
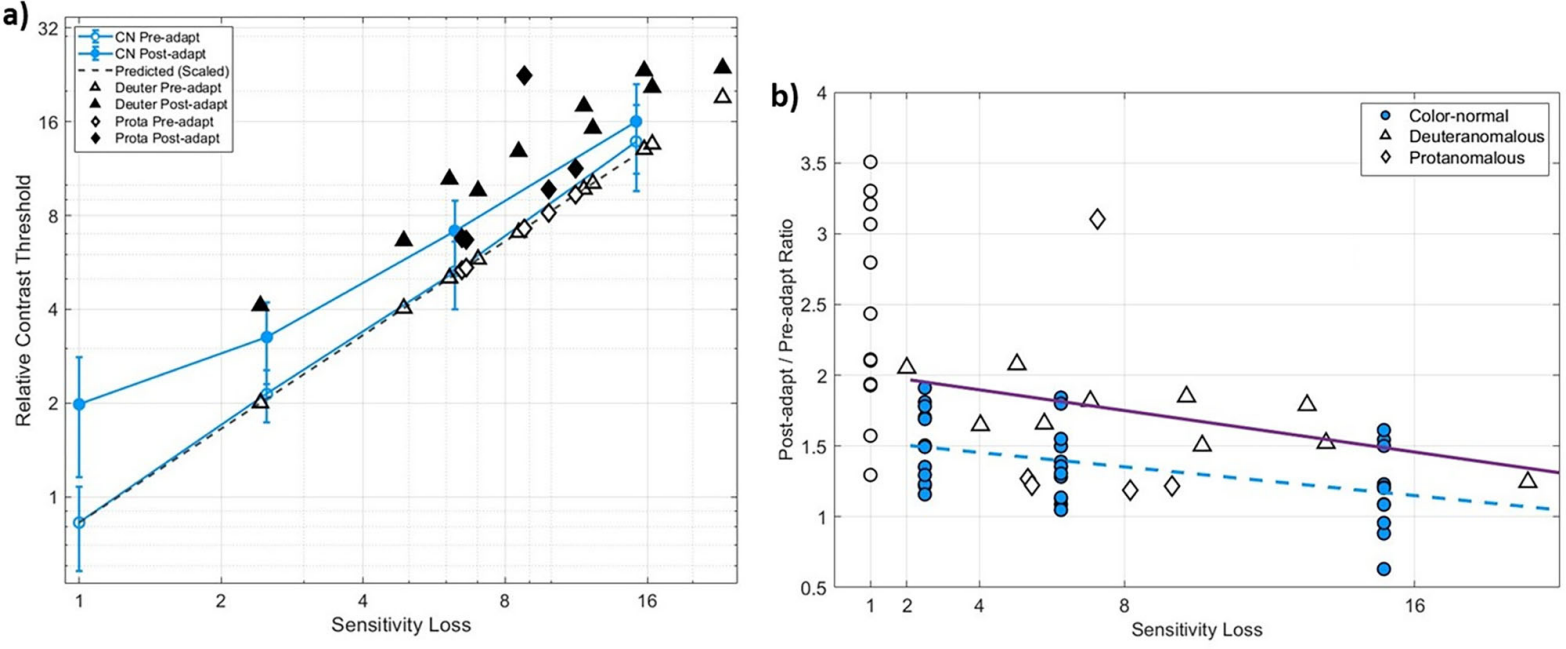
(**a**) Thresholds before (unfilled symbols) and after (filled symbols) adaptation for the individual anomalous trichromats (deuteranomalous: diamonds; protanomalous: triangles) plotted as a function of sensitivity loss. Blue lines and symbols show the predicted thresholds based on the stimulus rescaling scaling for color-normal thresholds, replotted from Figure 4a. (**b**) Magnitude of adaptation (post-adapt/pre-adapt threshold) for the different observers, plotted as a function of the scaled sensitivity loss for the color-normal observers (blue circles), or of the measured sensitivity loss for the deutan (unfilled triangles) or protan (unfilled diamonds) observers. Lines show the best-fitting regression lines to the color-normal (blue dashed line) or deutan (purple solid line) settings over the sensitivity range from 2 to 20 (thus excluding the unscaled CN values at 1).

This comparison is shown in Figure 5, which replots the thresholds before and after adaptation (Figure 5a) or the threshold ratios (Figure 5b) for the individual observers for each group. For the protan observers, the results are mixed. Four of the observers had threshold ratios slightly below the predicted values, suggesting little compensation. The remaining protanomalous participant had a greater than threefold change in threshold with adaptation, comparable to the stronger adaptation effects observed with unscaled CN ratios. The adapted thresholds for this observer were more variable (Figure 2, rightmost individual) but nevertheless strong across the two sessions despite their roughly fivefold weaker threshold sensitivity to the LvsM contrasts.

**Figure 6.**
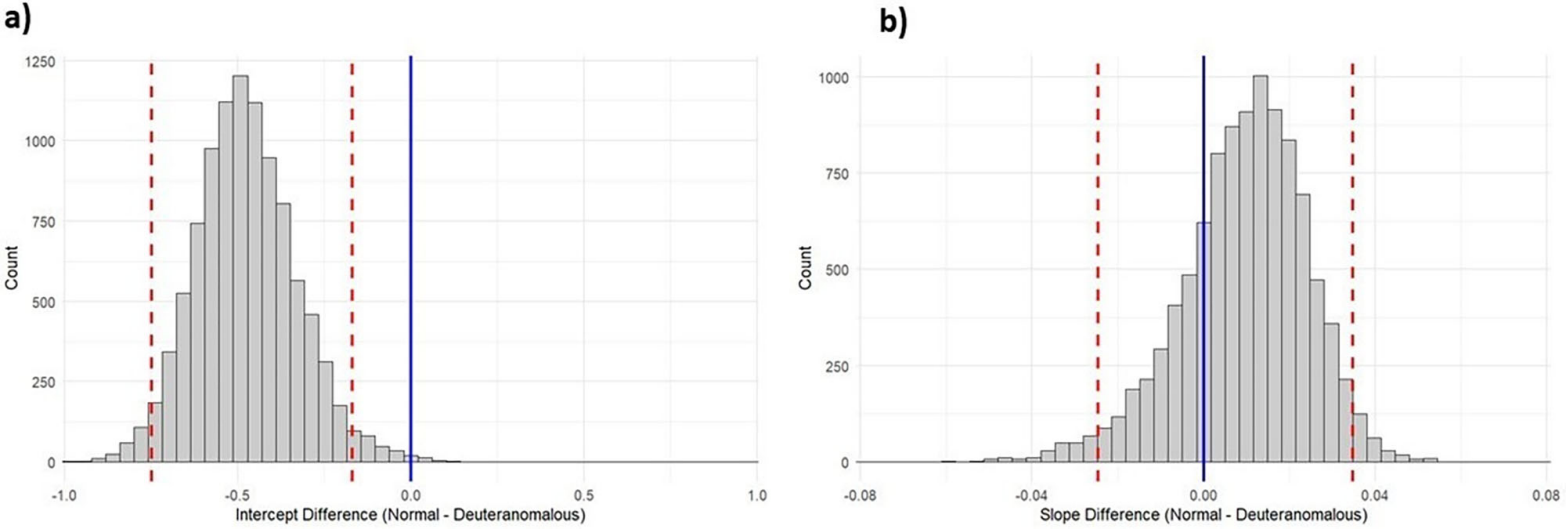
Bootstrap estimates of the differences in the intercepts (a) or slopes (b) of the regression lines fit to the normal vs. deutan observers. Dashed red lines are 95 percentile confidence intervals.

In comparison, for the 10 deutan observers, all of the observed post/pre-adapt threshold ratios were above the predicted no-compensation ratio for their respective sensitivities, a difference which is significant based on a simple sign test (*n* = 10, *p* = 0.002). To fully quantify the difference, we fit separate regression lines to the ratios for the deutan observers and to the values for the CNs at the three levels of reduced contrast scaling. (The unscaled values were not included for the CN fits, so that the fits could be compared over similar sensitivity ranges.) The regression lines are shown in Figure 5b. To compare the slopes and intercepts, we used a bootstrap resampling method with 10,000 iterations (Efron & Tibshirani, 1994). For this analysis, we repeatedly resampled participants within each group with replacement, re-ran the regression models, and calculated the differences in slopes and intercepts between groups in each iteration. The resulting bootstrap distributions were used to derive the 95 percentile confidence intervals for the slope and intercept differences (Figure 6). The confidence interval for the difference in slopes included zero, thus indicating the slopes did not differ significantly between the two groups. In other words, there was not a significant difference in how adaptation ratios changed with increasing sensitivity loss between the two groups, over the range of the AT sensitivities. In contrast, the confidence interval for the difference in intercepts did not include zero, indicating that the intercept of the deutan groups is significantly higher than that of the color-normal groups. This indicates that the deutan observers exhibited significantly higher adaptation levels compared to the settings for color-normal observers, again over the comparable sensitivity range. The higher intercept but similar slope is consistent with a similar multiplicative compensatory gain independent of the observer's sensitivity loss, though the specific pattern depends on a number of factors including nonlinearities in both the contrast response and adaptation.

A similar analysis (not shown) was also applied to the merged group of deutan and protan observers. This yielded a similar regression line as for the deutan observers alone, but one which was not significantly different in slope or intercept from the predicted sensitivity losses. Again this is because of the lower threshold ratios (and higher variance) for the protanomalous observers.

## Discussion

As noted in the Introduction, compensation for color vision in anomalous trichromacy has now been documented in a wide variety of tasks, but many questions remain about the nature and sites of the adjustments (Bosten, 2019; Isherwood et al., 2020). This includes whether the compensation reflects actual amplification of the post-receptoral signals or instead involves learning and decision rules for how colors are experienced and described. The pattern of adaptation we found suggests that in many color-anomalous observers, and deutan observers in particular, there is significant amplification of the neural LvsM color signals (at least in the circuits underlying contrast adaptation), so that the strength of the color signals are more similar to normal trichromats than predicted by the anomalous cone spectral sensitivities.

Our work complements other psychophysical studies designed to directly measure neural amplification of the color responses in anomalous trichromats. Boehm et al. (2021) compared contrast discrimination functions in CN and AT observers. Thresholds for detecting a contrast increment increase as the pedestal contrast increases, in similar ways for chromatic and luminance contrast (Switkes, Bradley, & De Valois, 1988), and this is thought to reflect a compressive nonlinearity in the response to contrast (Legge & Foley, 1980; von der Twer & MacLeod, 2001). ATs exhibited higher increment thresholds than predicted by their sensitivity losses, suggesting that their responses to the suprathreshold pedestals might be amplified relative to the contrasts predicted by their cone sensitivities. However, interpreting contrast discrimination is difficult because it depends on the properties of both the signal and the noise, and the results did not clearly establish the potential bases for the compensation. Similar to our study, Robinson, Bosten and MacLeod also used a cortical color adaptation effect to examine compensation in anomalous trichromacy (Robinson et al., 2023). In their case this involved measuring the strength of the McCollough Effect (a contingent aftereffect between color and orientation that is distinct from contrast adaptation; e.g., because it can have a very long persistence (Vul, Krizay, & MacLeod, 2008)). This study again took advantage of the compressive responses to color contrast, which for no compensation predict that increasing adaptor strength should lead to proportionally stronger effects in ATs, because responses to the adapting contrast should show less saturation. Matches to the aftereffects were best explained by a partial compensatory adjustment that boosted higher contrast signals (i.e., the strength of the adaptors), while having less effect at the low contrasts measured by matches to the aftereffects.

A common feature with many studies is the prediction that compensation is not manifest at threshold, where performance is limited by the intrinsic noise in the system. Color deficiencies are after all revealed by the lower sensitivity for detecting and discriminating colors, along with variations in the mean and range of color matches (as in the anomaloscope). As noted, Boehm et al. showed that the differences in the peak separation between the anomalous observers’ cones should be inversely proportional to their sensitivity (assuming fixed noise), and thus that sensitivity provides an estimate of the pigment shift (Boehm et al., 2014). However, above threshold, the visual system could potentially amplify the neural gain to recover responses to the weaker signal. This is seen in the responses to spatial contrast, where at threshold the contrast sensitivity function is bandpass, while above threshold perceived contrast becomes roughly independent of spatial frequency (Georgeson & Sullivan, 1975). However, how compensation is manifest in different perceptual tasks will depend in part on whether the amplification occurs before or after the sites of noise (Boehm et al., 2021). For example, if the signal and noise are both amplified, then color could appear more vivid yet discrimination remain poor (Webster, Juricevic, & McDermott, 2010). How this manifests in different individuals and conditions remains poorly understood. For example, some ATs show good discrimination despite the shifts in their pigments predicted from their Raleigh matches (Bosten, 2019).

The present results do not depend on specific assumptions about how compensation boosts the neural responses or the nonlinearities in those responses, except for the assumption that adaptation strength continues to increase monotonically with contrast, which held for the range of contrast levels we tested. The analyses also do not depend on the form of the response changes induced by adaptation. At higher test contrasts, contrast adaptation roughly follows a subtractive change in apparent contrast (Georgeson, 1985), but may more closely resemble a divisive change at lower test contrasts (Webster, 1996; Webster & Mollon, 1994). However, the proportional changes with adaptation that we assessed are based on predictions about how changes in the cone spectral peaks rescale the effective LvsM signals, and not on the specific ways in which these signals are coded or adapted.

Neural amplification has also been directly tested by imaging cortical responses to color in anomalous trichromats. Tregillus et al. (Tregillus et al., 2021) compared BOLD responses to chromatic contrast in CN and AT observers. In V1, responses were weaker for ATs, by roughly the amount predicted by their sensitivity losses (i.e., little or no compensation). However, in V2 and V3 the mean responses were similar across groups, suggesting amplification of the signals in V2, or possibly in the output of the responses inherited from V1.

The present results do not test the sites of the adaptation or compensation. However as outlined in the Introduction, several lines of psychophysical evidence suggest that chromatic contrast adaptation reflects response changes in the cortex. Physiologically, cells in the parvocellular pathway, the primary conduit for LvsM signals in the retina and geniculate, show relatively little adaptation to contrast (Solomon, Peirce, Dhruv, & Lennie, 2004). However, robust contrast adaptation effects are observed to color in V1 (Engel & Furmanski, 2001). Single-unit studies of color contrast adaptation have also suggested that a prominent component of the sensitivity change arises at early stages in V1 processing (Tailby, Solomon, Dhruv, & Lennie, 2008). Finally, an early cortical locus is also implied by the tuning of the behavioral aftereffects. Cells later in the cortical stream become progressively more narrowly tuned for color direction (Conway, Moeller, & Tsao, 2007; Kiper, Fenstemaker, & Gegenfurtner, 1997; Komatsu, Ideura, Kaji, & Yamane, 1992), with some narrowing evident even in V1 (De Valois, Cottaris, Elfar, Mahon, & Wilson, 2000). However, the psychophysical adaptation effects are consistent with the broader tuning predicted by different linear combinations of the cones (Webster & Mollon, 1991) (though the adaptation also shows some evidence for weak sensitivity changes in mechanisms more narrowly tuned for color direction (Mizokami, Paras, & Webster, 2004)). Together these results point to V1 as one probable site contributing to the adaptation and compensation, which as noted is earlier than the V2 locus suggested by fMRI responses to color in anomalous trichromats (Tregillus et al., 2021). However, the present adaptation results also suggest weaker levels of compensation than suggested by the measured BOLD responses in V2 and V3, which were close to the color normals. How the BOLD responses map onto the adaptation measured psychophysically remains uncertain (Krekelberg, Boynton, & van Wezel, 2006). In early visual cortex, adaptation in the BOLD responses does not show the strong selectivity for luminance vs. chromatic contrast evident in behavioral measurements, and thus may not directly reflect the neural underpinnings of the perceptual changes (Goddard, Chang, Hess, & Mullen, 2019). Alternately, MEG recordings do exhibit stronger parallels to the behavioral adaptation, and these also point to adjustments arising in early cortical stages (Goddard, Shooner, & Mullen, 2022). In any case, taken together, the properties of the aftereffects can plausibly be attributed to how color signals are represented and calibrated early in visual cortex.

Another source of uncertainty is that there are typically large individual differences in the degree of compensation found, which are not closely associated with the sensitivity losses (Bosten, 2019; Isherwood et al., 2020). The bases for these variations are also unknown. In this study we also observed a trend for compensation to be weaker in the protanomalous than deuteranomalous participants. We found a similar difference when a subset of the same observers were tested in a color naming task, where the protan observers reported achromatic percepts (“gray” or “white”) over a wider range of chromatic contrasts than the deutan observers (Basim et al., 2025). However, other studies provide only limited evidence of compensation differences between deutan and protan observers. Anecdotally, in the color salience measures of Regan and Mollon (1997), the two anomalous observers who performed most like color-normals were deuteranomalous. Boehm et al. (2014) found that color scaling for deutan observers tended to be more similar on average to color-normal observers, yet their average sensitivity losses were also less pronounced than for the protan group. Still other studies point to little differences in the two classes (e.g. Knoblauch et al. 2020). The source of any potential differences in compensation is unclear, since the spectral sensitivity differences in both cases result only from substitutions in the photopigment. One possibility is that our small sample is unrepresentative, and one of the protans did in fact have one of the largest measured aftereffects. Another possibility is that the threshold losses were underestimated for the protan observers. However, as Figure 5 shows, the adaptation in deutan participants was consistent with some compensation even for observers whose thresholds were many times higher.

Apart from its utility as a tool for probing visual responses, the effects of adaptation on color coding are also closely connected to both functional and mechanistic accounts of compensation for color deficiencies, and for sensory calibration in general. To efficiently encode information, the operating range of neural responses should be matched to the range of the available inputs. This argument successfully predicts how contrast coding is adapted to the distribution of contrasts in the environment, including the compressive nonlinearity in the contrast responses (Laughlin, 1981). It also provides a rationale for the higher gain in chromatic than luminance mechanisms, since the overlap of the cone spectral sensitivities results in a much smaller signal for color (e.g. LvsM) than luminance (L+M) (von der Twer & MacLeod, 2001). Thus normal color vision is already adapted to compensate for the enormous asymmetries between chromatic and luminance signals, and by this account, the adjustments expected for an anomalous trichromat are more a matter of degree than kind. The evidence that this adjustment primarily arises in the cortex is at first surprising, because it suggests that color coding in the retina and geniculate remains inefficient. However, the parvocellular pathway also carries achromatic information so that the operating range must be calibrated for both achromatic and chromatic inputs (Emery, Isherwood, & Webster, 2023; Lutze, Pokorny, & Smith, 2006). Selective compensation for chromatic sensitivity may therefore require how signals are recoded in the cortex.

In terms of potential mechanisms for compensation, the fact that adaptation can adjust selectively for the gamut of colors an observer is exposed to shows that these adjustments can in principle recalibrate color coding for differences in both environments and observers, and that these changes can occur rapidly. On the other hand, short-term adaptation is typically more robust for increases than decreases in contrast, which may nevertheless occur at longer timescales (Kwon, Legge, Fang, Cheong, & He, 2009). As a congenital trait, anomalous trichromats have had a lifetime to adapt, yet the compensation observed is often only partial and as noted can vary widely across individuals. The factors constraining compensation are an important unresolved question in understanding the nature and limits of sensory plasticity (Bosten, Coen-Cagli, Franklin, Solomon, & Webster, 2022). Nevertheless, our results add to a growing range of evidence suggesting that color perception in individuals with anomalous trichromacy is not as impoverished as their cone sensitivities predict, and add to further evidence that this adjustment includes actual sensory gains in the response to color.
